# Polycystic ovary syndrome and periodontal disease: an until-now undiscovered connection influenced by metabolic parameters

**DOI:** 10.1186/s12903-026-09196-4

**Published:** 2026-07-10

**Authors:** Cecilia Fabiana Márquez-Arrico, María Pelechá-Salvador, Sandra López-Domènech, Meylin Fernández-Reyes, Francisco Javier Silvestre, Javier Silvestre-Rangil, José María Montiel-Company, Milagros Rocha

**Affiliations:** 1 Department of Stomatology, University of Valencia, Gascó Oliag nº1, Valencia, 46010 Spain; 2 Department of Endocrinology and Nutrition, Foundation for the Promotion of Health and Biomedical Research in the Valencian Region (FISABIO), University Hospital Doctor Peset, Valencia, 46017 Spain

## Abstract

**Aims:**

This study aimed to determine whether women with polycystic ovary syndrome (PCOS) exhibit a higher prevalence of periodontal diseases (PD) than healthy and to evaluate the influence of potential confounders, including body mass index (BMI), abdominal obesity, biochemical markers, and oral hygiene habits, on periodontal status.

**Materials and methods:**

A cross-sectional analytical study was conducted at the University Hospital Dr. Peset in Valencia, Spain. The case group, aged 18–45 years and PCOS diagnosed according to the Rotterdam criteria, and controls were healthy women matched by age and BMI. All participants underwent periodontal examinations, biochemical and hormonal blood analyses, anthropometric assessments, and oral hygiene habits questionnaire.

**Results:**

PCOS group exhibited an altered metabolic profile, including elevated insulin levels, insulin resistance, dyslipidemia, and increased inflammatory markers, indicating a systemic pro-inflammatory state. PD were significantly more prevalent in PCOS than controls (40.5% vs. 11.2%), with higher rates of gingivitis and slightly increased periodontitis. PCOS showed greater bleeding on probing, plaque accumulation, and clinical attachment loss in PCOS. Correlation analyses revealed associations between PD and metabolic/inflammatory markers, including insulin, HDL cholesterol, hsCRP, C3c, and RBP4. Multivariate regression identified insulin and HDL cholesterol as independent predictors of periodontal inflammation, while C3c predicted plaque accumulation. CHAID analysis indicated fasting insulin as the strongest discriminator, with higher levels linked to increased PD prevalence.

**Conclusions:**

PCOS is independently associated with periodontal diseases, and this relationship appears mediated by metabolic and inflammatory alterations, with insulin resistance emerging as a central mechanistic link between systemic and oral health.

## Introduction

The association between systemic diseases and periodontal diseases (PDs) has increasingly become a focus of research. The chronic immune response triggered in periodontal tissues can extend to the bloodstream, contributing to a state of low-grade systemic inflammation. Inflammatory mediators produced in PDs – such as C-reactive protein (CRP), complement component C3 (C3c), interleukins (IL), and tumour necrosis factor α (TNFα) – can promote endothelial dysfunction and accelerate atherosclerosis [1]. Conversely, individuals with chronic inflammatory and metabolic conditions, such as diabetes, obesity, and metabolic syndrome, exhibit increased susceptibility to PDs, while periodontal inflammation itself can undermine glycaemic control, thus supporting the existence of a bidirectional relationship between periodontal and systemic diseases [2–4].

In recent years, an association between PDs and polycystic ovary syndrome (PCOS) has been proposed [5, 6].According to the World Health Organization (WHO), his syndrome affects 6–13% ofwomen of reproductive age worldwide and often remains an underdiagnosed pathology due to variability among the diagnostic criteria of different scientific societies [7]. Typically, PCOS manifests through the presence of clinical and/or biochemical hyperandrogenism, anovulation, or polycystic ovarian morphology. Beyond its reproductive manifestations, PCOS is increasingly recognized as a systemic disorder associated with oxidative stress and chronic low-grade inflammation, as well as heightened reactive oxygen species (ROS), lipid peroxidation markers, CRP levels, inflammatory cytokines, and elevated percentages of circulating lymphocytes and monocytes, alongside reduced antioxidant defences [8, 9].

PDs encompass a set of pathologies that affect the dental support tissues (periodontium), including gingivitis, characterised by inflammation confined to the gingiva and periodontitis, which, in addition to gingival inflammation, involves the progressive destruction of the periodontal ligament and alveolar bone [10]. The stimulation and secretion of proinflammatory cytokines associated with periodontitis contribute to insulin resistance (IR), a key pathophysiological feature also present in PCOS [11]. This shared inflammatory and metabolic milieu provides a plausible biological framework common to both conditions [3, 11, 12].

Despite this mechanistic rationale, hard evidence of the relationship between PCOS and PDs remains limited. Özçaka et al. reported that PCOS can exacerbate gingival inflammation through increased IL17 production, both in serum and in gingival crevicular fluid (GCF), with higher IL17 levels observed in women with PCOS and gingivitis versus controls [13]. Similarly, Porwal et al. described higher rates of gingival inflammation and periodontal destruction in women with PCOS, suggesting an increased susceptibility to gingivitis and subsequent progression to periodontitis in this population [12]. Recently, Pavankumar et al., 2023 [14] and Lui et al., 2024 [15] reported greater gingival inflammation and clinical attachment loss in a PCOS group compared to controls, thereby reaffirming the association.

Furthermore, metabolic disturbances commonly observed in PCOS, including alterations in lipid and carbohydrate metabolism, may further amplify systemic inflammation and IR, thereby reinforcing periodontal tissue breakdown and contributing to the convergence of both disease [3, 4, 16] .

The aim of this study was to determine whether women with PCOS exhibit a higher prevalence of PDs than controls and to evaluate the influence of confounding variables –body mass index (BMI), abdominal obesity, biochemical parameters, and oral habits – on periodontal status.

## Materials and methods

### Study design

**Fig. 1:**
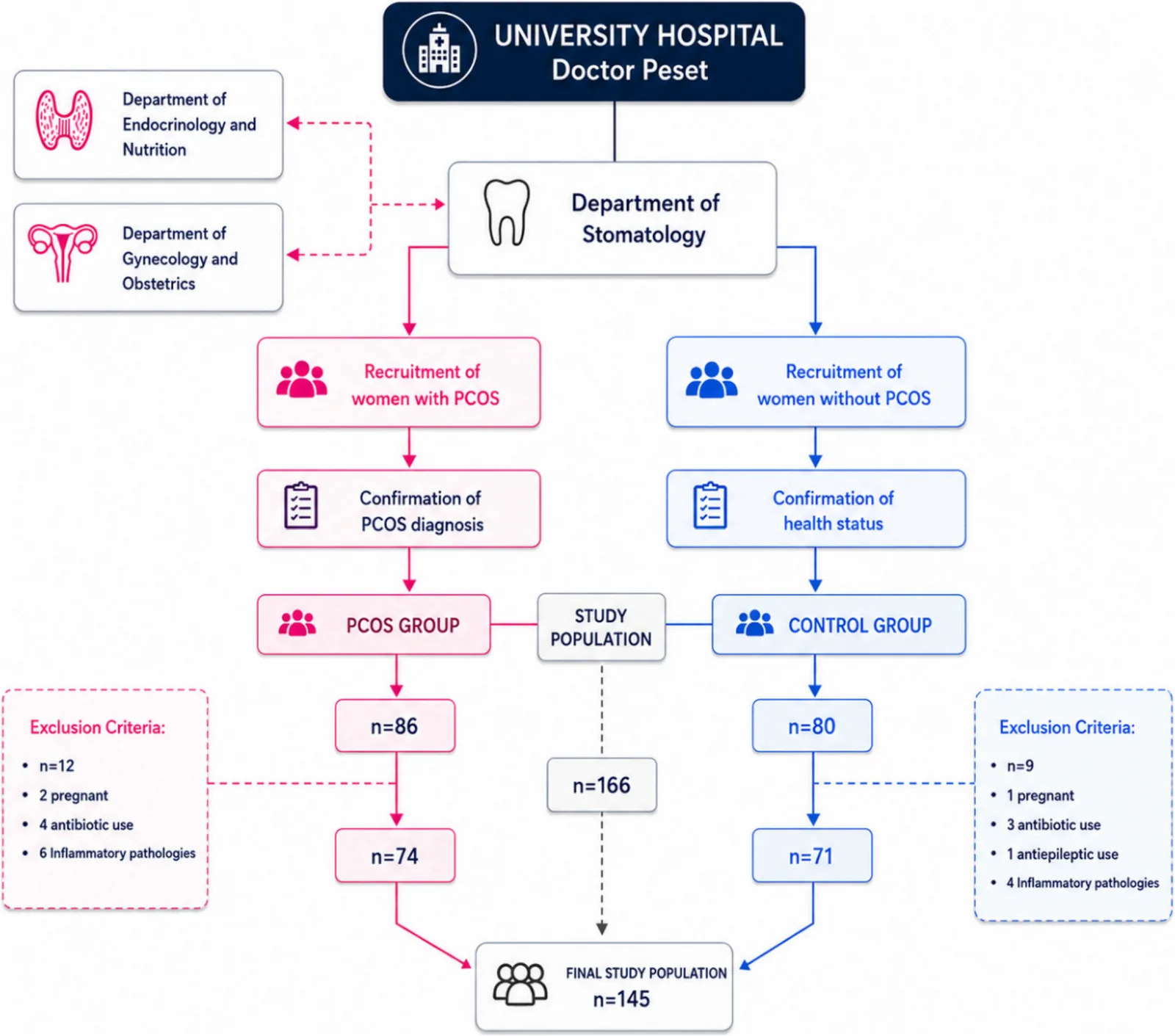
Flow diagram of participant recruitment, eligibility assessment and inclusion in the study

A cross-sectional analytical study was conducted at Dr. Peset University Hospital, Valencia, Spain. Women aged 18–45 years and diagnosed with PCOS according to the Rotterdam criteria [17] were recruited and compared with age-matched women without PCOS or other relevant medical conditions. Use of medication, comorbidities, and other clinical characteristics were recorded in both groups. Exclusion criteria included the presence of inflammatory diseases, antibiotic therapy within the previous 6 months, recent or chronic use of anti-inflammatory drugs, cancer, bone-related disorders, diabetes, autoimmune diseases, and other conditions known to affect periodontal health. A total of 145 women were included in the study: 74 with PCOS and 71 controls (Fig. 1).

All procedures were approved by the “Research Ethics Committee for Medicines” (CEIm) of the Doctor Peset University Hospital in Valencia (approval document nº Ceim 31/19, registered under NCT06184412 on 2020/02/01). Written informed consent was obtained from all participants after providing them with detailed information about the study.

### Clinical examination and biochemical and periodontal parameters

Data collection followed a standardized protocol. All participants completed a structured health questionnaire that collected information regarding medical history, current medication use, tobacco and alcohol consumption, oral hygiene habits (using the WHO Oral Health Questionnaire for Adults [18]) and blood pressure. Anthropometric measurements included body weight (kg), height (m), and waist and hip circumferences (cm). BMI, waist-to-hip ratio (WHR), and waist-to-stature ratio (WSR) were calculated using standardized formulas. Hormonal contraceptive use (type and duration) and metformin treatment were recorded and considered as potential confounding variables.

### Periodontal examination

A comprehensive periodontal examination was performed by a single calibrated examiner trained in periodontics (C.F.M.A.), in accordance with the Consensus Report of Workgroup 2 of the 2017 World Workshop on the Classification of Periodontal and Peri-Implant Diseases and Conditions [19]. All teeth present were examined using a Williams-type millimetre periodontal probe, with six sites assessed per tooth (three buccal and three palatal/lingual), and data were recorded in a periodontal chart, thereby avoiding inter-examiner variability. The periodontal parameters evaluated included probing pocket depth (PPD), clinical attachment loss (CAL), and bleeding on probing (BOP). The number of teeth with PPD ≥ 4 mm and CAL ≥ 4 mm was also recorded, as was percentage of bone loss. Plaque accumulation was assessed using the Silness and Löe plaque index, and calculus accumulation was evaluated using the O’Leary index [20]. For feasibility reasons related to the high patient flow in our hospital setting, plaque and calculus indices were measured on the Ramfjord index teeth [21]. Information on tobacco use, tooth brushing frequency, alcohol consumption, and previous dental treatments – particularly periodontal therapy and orthodontic procedures potentially associated with gingival recession – was collected through a structured questionnaire. Radiographic assessment included panoramic radiography for all participants, with additional periapical radiographs obtained in cases where periodontal pockets were detected.

### Blood sampling

Peripheral venous blood samples were obtained after 12-hour overnight fasting between days 1 and 5 of menstrual bleeding, either during spontaneous menstruation or while using combined oral contraceptives (OCs), in order to minimize variability related to sex hormone fluctuations. Analyses included sex hormone profiles, lipid profile, and parameters of carbohydrate metabolism, all determined at the hospital’s Clinical Analysis Service using standardized and validated protocols, as described previously [22]. The Homeostatic Model Assessment of Insulin Resistance (HOMA-IR) was calculated by multiplying fasting insulin (mIU/mL) by glucose (mg/dL) and dividing by 405. Values > 3.0 indicate IR.

Analysis of circulating inflammatory cytokine levels and TNFα levels were measured in serum with a bead-based immunoassay (Milliplex MAP; Millipore Corporation, Billerica, MA, USA) and the Luminex^®^ 200 analyser system (Luminex Thermo Fisher Scientific, Waltham, MA, USA), following the manufacturer’s procedure.

### Statistical analysis

It was estimated that a sample of 90 patients would be sufficient to determine differences between PCOS and controls (for the determination of means of two independent samples), accepting an alpha risk of 0.05 and a beta risk of 0.2 in a two-sided test. Forty-five subjects in the first group and forty-five in the second were required to detect a difference equal to or greater than 1 unit. A common standard deviation (SD) of 1.5 mm or less was assumed based on the following factors: the probe in question determines differences of 1 mm for both PPD and CAL; a single, properly calibrated explorer performed measurements; and previous studies conducted by our research group by the authors Pelechá-Salvador M. et al., 2025; Márquez-Arrico CF et al., 2025; Martinez-Herrera M et al., 2027 [22–24] yielded an SD of less than 1 for periodontal variables. A loss-to-follow-up rate of 20% has been estimated.

The sample size was calculated using the following formula:

$$n=\frac{2\sigma^2\left(Z_{\alpha/2}+Z_\beta\right)^2}{\triangle^2}$$

Where n is the number of subjects per group, σ is the common standard deviation, Δ is the minimum difference to be detected, Zα/_2_ corresponds to the selected significance level, and Z_β_ corresponds to the desired statistical power.

To determine the normality of the data, the Shapiro-Wilk and Kolmogorov-Smirnov normality tests were applied. Descriptive statistics are presented as means and percentages for normally distributed variables, and as medians with interquartile ranges (25th–75th percentiles) for non-normally distributed variables, according to group (PCOS vs. control). Inferential analyses included the Chi-square test for comparisons of categorical variables, Student’s t-test and one-way ANOVA with Bonferroni post hoc correction for normally distributed continuous variables, and the Mann-Whitney U test and Kruskal-Wallis test with Bonferroni-adjusted pairwise comparisons for non-normally distributed variables.

Odds ratios (OR) were calculated to estimate the probability of presenting periodontal diseases associated with PCOS status, using dichotomized periodontal outcomes (periodontal health vs. gingivitis and/or periodontitis). Multivariable logistic regression models were applied including variables that showed significant differences between groups. Additionally, linear, binary logistic, and multinomial regression models were used to assess the predictive contribution of the studied parameters to PCOS status and periodontal disease outcomes. Associations between continuous variables with non-normal distribution were assessed using Spearman’s rank correlation. Finally, Chi-square automatic interaction detection (CHAID) decision tree analysis was performed to identify variables most strongly associated with periodontal disease outcomes and to explore potential interactions between predictors related to PCOS status. The model allowed the classification of participants according to periodontal condition based on significant splitting variables.

Analysis was performed with SPSS 22.0 (SPSS Statistics Inc., Chicago, IL, USA). Differences were deemed significant when *p* < 0.05, with a 95% confidence interval.

## Results

One hundred and forty-five patients were analysed, with a mean age of 27.9 years in the PCOS group and 28.1 years in the control group (*p* = 0.844). Most participants had a high level of educational attainment, with 52.7% of the PCOS group and 45.0% of the controls holding a university degree, and with no significant differences between PCOS and controls in academic qualifications across the educational categories. (*p* = 0.097) (Table 1). Employment status did not differ significantly between groups (*p* = 0.418) (Table 1), with 62.1% of the PCOS group and 70.4% of controls in employment, 33.7% and 23.9% students, and 4.2% and 5.7% unemployed, respectively. In general, the whole population had good oral hygiene habits, with most of them brushing their teeth more than twice a day. However, there were differences between the oral hygiene habits of the PCOS group and those of controls; of special relevance was the frequency of tooth brushing of only once per day in the PCOS group (14.8%) compared to that in the control group (V) (*p* = 0.031) (Table 1). There were no significant differences in the use of interdental hygiene elements (interproximal brushes, floss and interdental arches) between the PCOS group and controls. The frequency of visits to the dentist was similar among PCOS and controls, with the majority having attended their last appointment less than a year ago, and without significant differences being detected (Table 1). Alcohol and tobacco consumption was similar in both groups, again without significant differences.

**Table 1:** Descriptive analysis of sociodemographic parameters, oral hygiene habits, and toxic habits comparing the PCOS group with the control group

		Control (*n* = 71)	PCOS (*n* = 74)
Age (years)		28.1 ± 5.91	27.9 ± 5.66
Educational level, % (n)	Degree/Grade	45.0 (32)	52.7 (39)
	Master’s/Doctorate	19.7 (14)	8.1 (6)
	Pre-University Secondary education/AVT	23.9 (17)	28.4 (21)
	CSE/IVT	2.81 (2)	8.1 (6)
	Basic	4.22 (3)	2.7 (2)
Occupation, % (n)	Employed	70.4 (50)	62.1 (46)
	Student	23.9 (17)	33.7 (25)
	Unemployed	5.7 (4)	4.2 (3)
Tooth brushing frequency, % (n)	≥ 2 times a day	94.3 (67)	78.3 (58)
	Once a day	1.40 (1)	14.8 (11)*
	< Once a day	4.22 (3)	6.75 (5)
Interdental hygiene, % (n)	Yes	47.8 (34)	40.5 (30)
	No	52.1 (37)	59.4 (44)
Alcohol consumption, % (n)	Yes	35.2 (25)	35.1 (26)
	No	64.7 (46)	64.8 (48)
Daily alcohol consumption %, (n)	< 1 drink	32.4 (23)	22.9 (17)
	2 drinks	4.22 (3)	8.10 (6)
	3 drinks	0 (0)	2.70 (2)
	4 drinks	0 (0)	1.35 (1)
	≥ 5 drinks	0 (0)	2.70 (2)
	No drink (30days)	63.3(45)	62.1 (46)
Tobacco, % (n)	Yes	16.9 (12)	21.6 (16)
	No	74.6 (53)	66.2 (49)
	Ex-smokers	8.5 (6)	12.2 (9)

Data are presented as mean ± SD for parametric variables or as median and range of quartiles (25% and 75% percentile) for non-parametric variables. A Student’s T was used for independent samples or a Mann Witney U for independent samples according to the distribution of the variable, and the χ^2^ test was used for proportions. *p < 0.05; **p < 0.01;*** *p* < 0.001

*Abbreviations:* *CSE* Compulsory secondary education, *VT* Vocational training (A: advance, I: intermediate), *PCOS* Polycystic ovary syndrome

Next, we evaluated anthropometric and biochemical characteristics in order to characterize the study population. Despite a lack of differences in age, women with PCOS exhibited significantly higher BMI, waist circumference, WSR, and WHR compared with controls, thus indicating a more pronounced central adiposity. These changes were associated with higher diastolic blood pressure (Table 2). Biochemical analysis revealed that women with PCOS had significantly higher insulin levels and insulin resistance. Lipid profile analysis showed increased total cholesterol, LDLc, and triglycerides, along with reduced HDLc in women with PCOS. Inflammatory markers, including hsCRP, TNFα, C3c, and retinol-binding protein 4 (RBP4), were significantly elevated in the PCOS group, which reflected a proinflammatory state. Regarding sex hormones, women with PCOS had significantly lower estradiol and 17OH-progesterone levels, whereas follicle-stimulating hormone (FSH), luteinizing hormone (LH), testosterone, androstenedione, dehydroepiandrosterone sulfate (DHEAS), sex hormone-binding globulin (SHBG), free androgen index (IAL), and prolactin did not differ significantly from controls (Table 2). Overall, these findings indicate that women with PCOS present a metabolic profile characterized by central adiposity, insulin resistance, dyslipidemia, and systemic inflammation, accompanied by specific alterations in steroid hormones. As expected, the proportion of participants receiving pharmacological treatment – specifically, metformin and COCs - was higher in the PCOS group (Table 2).

**Table 2:** Descriptive and inferential analysis of biochemical variables, sex hormones, anthropometry and blood pressure comparing the PCOS group with the control group

	Control (*n* = 71)	PCOS (*n* = 74)
BMI (kg/m2)	22.0 (22.2, 24.6)	24.5 (21.3, 30.1)***
Waist (cm)	70 (67, 76 9)	77 (71,90)***
WSR	0.410 (0.390, 0.460)	0.470 (0.420, 0.542)***
WHR	0.740 (0.700, 0.760)	0.760 (0.727, 0.800)***
SBP (mm Hg)	109 (102, 116)	110 (103, 117)
DBP (mm Hg)	75 (70, 80)	77 (72, 85)*
Glucose (mg/dL)	85.0 ± 7.7	87.0 ± 12.6
Insulin (µUI/mL)	6.8 ± 1.8	10.0 ± 5.6***
HOMA-IR	1.43 ± 0.57	2.28 ± 1.82***
A1c (%)	5.03 ± 0.29	5.14 ± 0.22*
Cholesterol (mg/dL)	176 (155, 190)	187 (167, 213)**
HDLc (mg/dL)	64.6 ± 33.4	59.6 ± 31.1*
LDLc (mg/dL)	99.0 ± 14.3	112.8 ± 16.6**
Triglycerides (mg/dL)	65.0 ± 27.2	92.5 ± 47.2***
hsCRP (mg/L)	0.73 (0.32, 2.63)	3.71 (0.99, 7.06)***
TNFα (pg/mL)	11.0 ± 4.6	13.1 ± 3.7*
Fibrinogen (mg/dL)	404 ± 43	428 ± 117
C3c (mg/dL)	104 ± 44	126 ± 19***
Homocysteine (µmol/L)	9.46 ± 1.48	10.13 ± 9.86
RBP4 (mg/dL)	1.84 ± 0.47	2.19 ± 0.55***
FSH (mIU/dL)	3.90 (2.60, 5.00)	4.10 (1.95, 5.42)
LH (mUI/dL)	3.90 (2.80, 5.80)	3.70 (1.70, 5.62)
Estradiol (pg/mL)	55.0 (23.0, 136.0)	32.5 (10.0, 60.5)***
Testosterone (ng/dL)	0.404 ± 0.202	0.432 ± 0.187
Androstendione (ng/mL)	3.20 ± 1.22	3.71 ± 4.56
DEAHS (µg/dL)	276 ± 346	285 ± 124
17OH (ng/dL)	1.29 (0.79. 2.23)	0.80 (0.57. 1.19)***
SHBG(µg/dL)	74.0 (57.0. 95.0)	93.5 (39.7. 192.7)
IAL (%)	1.87 ± 1.08	2.51 ± 2.30
Prolactin (ng/dL).	21.7 ± 10.7	19.7 ± 10.9
Medication		
Metformin, % (n)	0 (0)	14.8 (11)***
COCs, % (n)	11.2 (8)	52.7 (39)***

Data are expressed as mean ± SD for parametric variables and as median and interquartiles 25 and 75 for non-parametric variables. A Student’s T was used for independent samples or a Mann Witney U for independent samples according to the distribution of the variable and the χ^2^ test for proportions. *p < 0.05; **p < 0.01; ***p < 0.001

*Abbreviations:* *17OH* 17-hydroxyprogesterone, *A1c%* percentage of glycated hemoglobin, *BMI* Body mass index, *BPD* Diastolic blood pressure, *C3* Complement 3, *COC* Combined oral contraceptives, *DEAHS* Dehydroepiandrosterone sulfate, *FSH* Follicle-stimulating hormone, *HDLc* High-density cholesterol, *HOMA-IR* Homeostatic model assessment of insulin resistance, *hsCRP* ultra-sensitive C-reactive protein, *IAL* free androgen index (percentage), *LDLc* Low-density cholesterol, *LH* Luteinizing hormone, *PCOS* Polycystic ovary syndrome, *RBP4* Retinol-binding protein A, *RI* dichotomous insulin resistance (IR = HOMA ≥ 3), *SBP* Systolic blood pressure, *SHBG* Sex hormone-binding globulin, *WHR* Waist-to-height ratio, *WSR* Waist/hip ratio

Regarding the presence of PDs, 38 women (26.3%) recorded the presence of some type of PDs, while the remaining 107 patients (73.7%) were periodontally healthy. Among those presenting PDs, 30 were PCOS (40.5%) and 8 were controls (11.2%), with a significantly higher prevalence of PDs in the PCOS group (*p* < 0.0001) (OR = 5.36, 95% CI 2.25–12.81). In an attempt to characterize the periodontal status of the study population, women with PCOS exhibited a significantly higher prevalence of gingivitis (32.3% vs. 8.5% in the control group, *p* < 0.001). While the prevalence of stage II and III periodontitis was low in both groups, women with PCOS showed slightly higher rates of localized and generalized periodontitis (Table 3).

**Table 3:** Descriptive and inferential analysis of periodontal status with respect to the PCOS and control group

	Control (*n* = 71)			PCOS (*n* = 74)
Classification of PDs, % (*n*)				
Healthy		88.7 (63)	59,5 (44)***	
Gingivitis		8.5 (6)	32.3 (24)***	
Periodontitis S I		0 (0)	1.4 (1)	
Periodontitis S II		2.8 (2)	5.4 (4)	
Periodontitis S III		0 (0)	1.4 (1)	
Extension of Periodontitis, % (n)				
Localized	1.40 (1)			6.75 (5)
Generalized	1.40 (1)			1.35 (1)
Periodontogram				
PPD (mm)	1.51 (1.35, 1.63)			1.58 (1.39, 1.82)
CAL (mm)	1.51 (1.35, 1.64)			1.58 (1.41, 1.82)
BOP (%)	1.78 (0.00, 4.46)			5.35 (1.83, 11.6)***
Bone loss (%)	0.952 ± 4.56			2.140 ± 7.290
Plaque Index	0.445 ± 0.452			0.728 ± 5.26*
Calculus Index	0.075 ± 0.276			0.089 ± 0.278
nº. of dental absences	0.394 ± 1.380			0.554 ± 1.240
nº. of PPD+ teeth	0.630 ± 2.52			0.930 ± 2.47
nº of CAL+ teeth	0.080 ± 0.405			0.470 ± 1.29*
Time since last dental visit, % (n)				
1–5 years	29.5 (21)			31.0 (23)
< 1 year	63.3 (45)			59.4 (44)
≥ 5 years	7.04 (5)			9.45 (7)
Treatment, % (n)				
Never	21.1 (15)			13.6 (10)
Tartrectomy	78.9 (56)			78.3 (58)
SRP	0 (0)			8.1 (6)*

Data are expressed as mean ± SD for parametric variables and as median and interquartile 25 and 75 for non-parametric variables. A Student’s T was used for independent samples or a Mann Witney U for independent samples according to the distribution of the variable and the χ^2^ test for proportions. * *p* < 0.05; **p < 0.01; ***p < 0.001

*Abbreviations:* *BOP%* Percentage of bleeding on probing, *CAL* Clinical attachment loss, *CAL+* Clinical attachment loss ≥ 4 mm, *S* Stage, *PCOS* Polycystic ovary syndrome, *PDs* Periodontal diseases, *PPD* Probing pocket depth, *PPD+* Probing pocket depth ≥4 mm, *S* Stage, *SRP* Scaling and root planning

Periodontal parameters revealed significantly higher BOP (*p* < 0.001), plaque index in the PCOS group (*p* < 0.05) and number of teeth with CAL ≥ 4 mm (*p* < 0.05). No significant differences were observed in PPD, bone loss, calculus index, number of missing teeth or number of teeth with PPD ≥ 4 mm between groups. Regarding prior periodontal treatment, a small proportion of women with PCOS had undergone scaling and root planning (SRP), which was significantly higher than in controls, whereas rates of tartrectomy were similar in both groups (Table 3). Overall, these findings indicate that women with PCOS present a worse periodontal profile characterized by increased gingival inflammation, higher plaque accumulation, and greater CAL, despite a similar history of prior treatment with respect to controls.

**Fig. 2:**
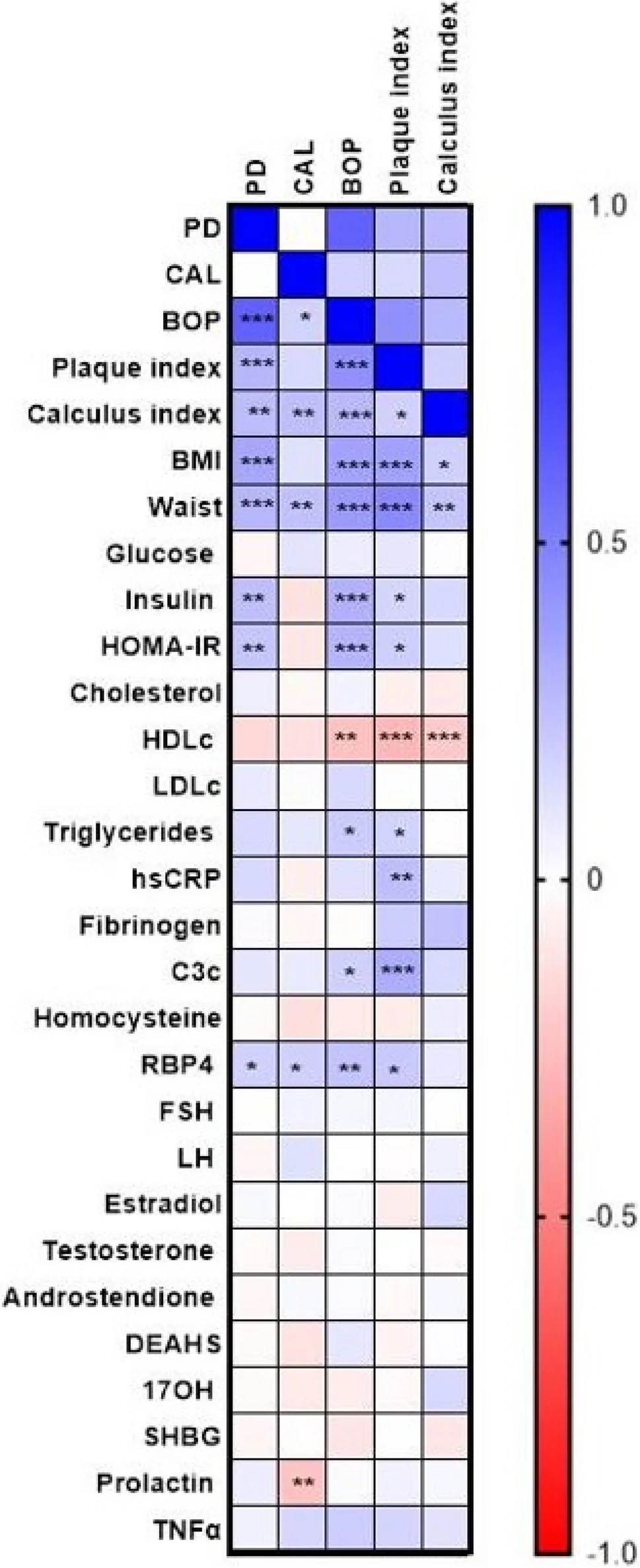
Heatmap of Spearman’s correlation coefficient matrix depicting the strength of relationships between periodontal parameters, anthropometric indices, and biochemical parameters. Negative correlations are shown in red while positive correlations are shown in blue. The darker the color, the stronger the correlation. ∗p < 0.05, ∗∗p < 0.01, ∗∗∗p < 0.001. BMI,; C3c, complement factor C3; HDLc, high-density lipoprotein cholesterol; HOMA-IR, homeostasis model assessment of insulin resistance; hsCRP, high-sensitivity C-reactive protein; LDLc, low-density lipoprotein cholesterol; TC, Total cholesterol TG, Triglycerides; BOP, bleeding on probing; CAL, clinical attachment lossl; PPD, probing pockets depth; RBP4, retinol-binding protein 4, FSH, Follicle-stimulating hormone, LH, luteinizing hormone, DEAHS, dehydroepiandrosterone sulfate, 17OH, 17-OH progesterone, SHBG, sex hormone binding globulin

Given that PCOS has been associated with poorer oral hygiene (Table 1), which in our study was reflected by a higher plaque index (Table 3), we performed a logistic regression analysis to determine whether the observed differences in oral hygiene could account for the increased prevalence of PDs in our population. The results indicated that, after adjustment for oral hygiene, belonging to the PCOS group remained independently associated with the presence of PDs (OR = 4.83, 95% CI: 1.99–11.73; *p* = 0.001). In contrast, oral hygiene showed an inverse association with PDs, acting as a protective factor (OR = 0.43, 95% CI: 0.21–0.86; *p* = 0.017) (Table 4). Subsequently, we evaluated potential associations between biochemical and periodontal parameters in order to identify possible mediators underlying the increased prevalence of periodontal diseases in women with PCOS. Waist circumference showed positive correlations with all periodontal parameters, while BMI was positively associated with most periodontal variables, except for CAL. Insulin levels and HOMA-IR were positively correlated with PPD, BOP, and plaque index. In contrast, HDLc was negatively correlated with BOP, plaque, and calculus indices, whereas triglycerides were positively associated with BOP and plaque accumulation. Inflammatory markers showed relevant associations, with hsCRP correlating positively with plaque index and C3c correlating with both BOP and plaque. RBP4 exhibited positive correlations with PD, CAL, BOP, plaque, and calculus indices. Finally, prolactin levels were negatively correlated with CAL (Fig. 2).

**Table 4:** Multivariate logistic regression model (stepwise method) for predictors of periodontal disease in women with PCOS and controls

Variable	B	SE	Wald	df	*p*-value	OR (Exp(B))	95% CI for OR
Group	1.574	0.453	12.077	1	0.001	4.83	1.99–11.73
Oral hygiene	-0.847	0.356	5.657	1	0.017	0.43	0.21–0.86
Constant	-0.387	1.579	0.060	1	0.806	0.68	–

The stepwise method is an automated statistical technique used in regression analysis to build a predictive model by iteratively adding or removing candidate predictor variables. In this case, the elimination method was used to introduce variables that influence periodontal pathology and that differed significantly significant differences between PCOS and controls. Oral hygiene was a protective factor against the presence of periodontal diseases. The association between PCOS and periodontal disease remained statistically significant after adjustment for oral hygiene

In order to identify the biochemical variables that contributed to periodontal parameters, a univariate regression analysis using a stepwise method was performed to evaluate independent predictors of periodontal outcomes (Table 5). Insulin levels were independently associated with probing pocket depth (PPD), explaining 8.3% of its variability. BOP was influenced by insulin and HDLc, jointly accounting for 12.2% of the observed variation, whereas plaque index was primarily associated with C3c, explaining 9.0% of its variability. In contrast, CAL and calculus index were not independently associated with any of the biochemical variables included in the model. These findings indicate that specific metabolic and inflammatory markers, particularly insulin and lipid-related parameters, are selectively associated with periodontal inflammation and plaque accumulation rather than with irreversible periodontal tissue damage.

**Table 5:** Univariate linear regression model of periodontal parameters and biochemical variables. Univariate linear regression model to evaluate the relationship between biochemical variables that showed significant differences between PCOS and controls and their relationship with periodontal parameters, adjusting the model with the covariate BMI

Dependent variable	Independent variable	β	Standard error	Beta coefficient	Adjust$\:{R}^{2}$	*p* value
PPD					0.083	0.017
	Constant	1.142	0.171			0.000
	Insulin	0.465	0.189	0.315		0.017
BOP					0.122	< 0.001
	Constant	17.711	9.203			0.056
	Insulin	6.078	2.139	0.247		0.005
	HDLc	-10.035	4.645	-0.188		0.032
Plaque index					0.090	< 0.001
	Constant	-2.820	0.889	0.311		0.002
	C3c	1.666	0.432	0.311		< 0.001

*Abbreviations:* *BOP* Bleeding on probing, *C3c* Complement factor C3, *HDLc* High density lipoprotein cholesterol, *PPD* Probing pocket depth

**Fig. 3:**
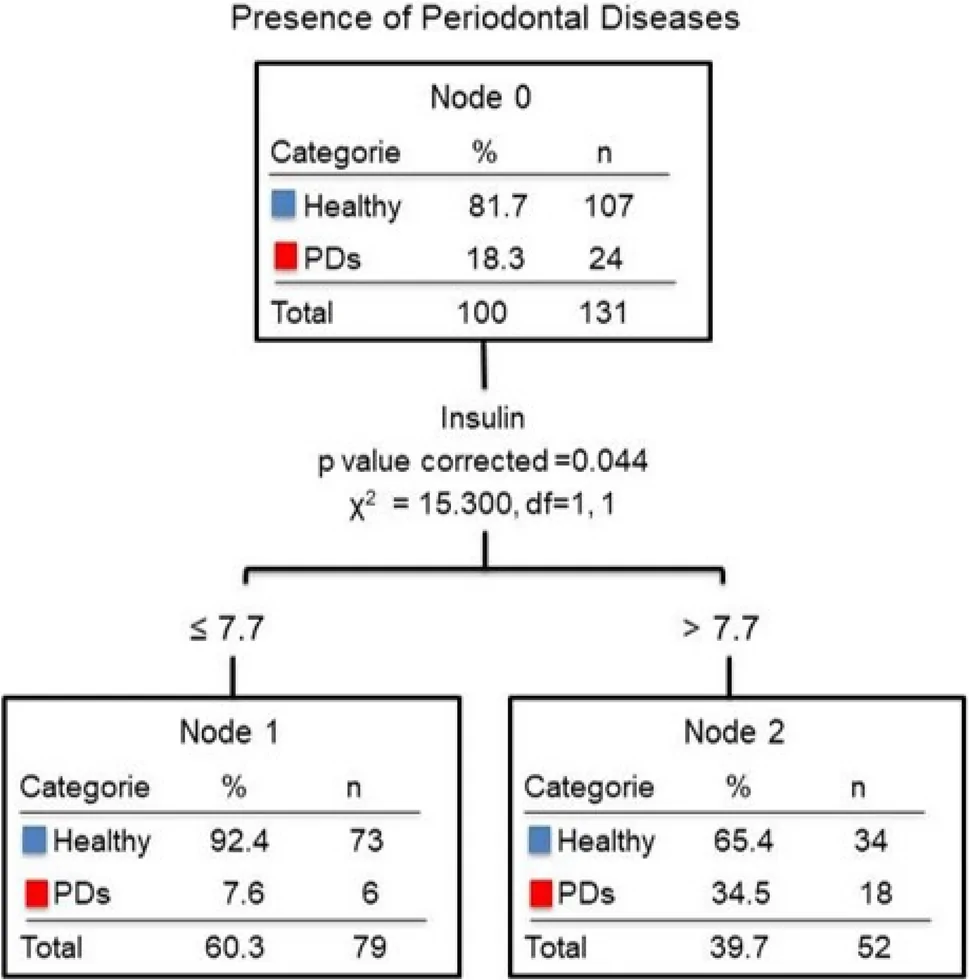
CHAID tree showing the relationship between fasting insulin and periodontal status. All variables that showed significant correlations with the presence of PD were included in the CHAID tree analysis. The findings suggest that fasting insulin was the variable with the greatest discriminative capacity with respect to periodontal status in the study population. Participants with insulin levels above 7.7 µIU/mL(cut-off = 7.7 µIU/mL, *p* = 0.044) showed a higher prevalence of periodontal disease compared with those presenting lower insulin concentrations

Finally, given the relevance of insulin in periodontal parameters observed in the multivariable regression analysis, we further explored its discriminative capacity using a CHAID analysis (Fig. 3). This approach identified fasting insulin as the variable that best distinguished participants with healthy periodontal status (Healthy) from those with PDs. In the total population, 81.7% were periodontally healthy, while 18.3% had PDs. The CHAID tree generated a root node that was split based on fasting insulin, with a cut-off of 7.7 µIU/mL (*p* = 0.044). Among participants with insulin ≤ 7.7 µIU/mL (Node 1, *n* = 79; 60.3% of the sample), the majority were periodontally healthy (92.4%, *n* = 73), and only 7.6% (*n* = 6) had PDs. In contrast, participants with insulin > 7.7 µIU/mL (Node 2, *n* = 52; 39.7% of the sample) had a higher prevalence of PDs (34.5%, *n* = 18), while 65.4% (*n* = 34) remained healthy. These findings indicate that higher fasting levels of insulin are associated with an increased risk of periodontal disease, whereas lower insulin levels are linked to a lower prevalence of PDs.

## Discussion

In this study, women with PCOS exhibited a significantly higher prevalence of PDs compared with age-matched controls, an association that was independent of oral hygiene habits. This greater susceptibility was driven mainly by a higher frequency of gingivitis and a poorer periodontal inflammatory profile characterized by increased bleeding on probing, and by greater plaque accumulation [23]. Beyond local oral factors, PCOS was associated with an evidently unfavourable metabolic and inflammatory profile marked by central adiposity, insulin resistance, dyslipidemia, and elevated systemic inflammatory markers, all of which were closely related to periodontal parameters [8, 15]. Notably, insulin emerged as a key determinant of periodontal inflammation, representing an independent predictor of probing depth and bleeding on probing with the capacity to identify individuals at higher risk of periodontal disease [24]. Considered together, these findings support a strong link between PCOS and periodontal disease that appears to be mediated, at least in part, by metabolic and inflammatory alterations rather than by oral hygiene alone.

Previous studies have identified PCOS as a potential determinant of PDs [12, 13, 25, 26], based on the increase in low-grade systemic inflammation that characterizes it, which further exacerbates the periodontium’s response to aggression from periodontopathogens. This inflammation has been demonstrated by an increase in IL17 in GCF in PCOS with gingivitis [13], and increases in BOP and CAL in PCOS patients compared to controls [12, 25–27]. Additionally, systemic antibody responses to these pathogens are heightened in PCOS, suggesting a broader immune activation that could further exacerbate periodontal inflammation regardless of BMI [26].

Oral hygiene habits are a well-recognized determinant of periodontal health and represent a potential confounding factor when evaluating periodontal outcomes [28]. In the present study, although oral hygiene habits were good overall, women with PCOS showed subtle but significant differences with respect to controls; particularly, a lower frequency of tooth brushing and a higher plaque index. Importantly, adjustment for oral hygiene did not abolish the association between PCOS and periodontal diseases; belonging to the PCOS group was independently associated with periodontal disease, while oral hygiene itself acted as a protective factor. Collectively, these findings indicate that poorer oral hygiene alone does not fully account for the increased periodontal burden in women with PCOS, suggesting that factors intrinsic to PCOS contribute to periodontal susceptibility beyond behavioural determinants [25, 26].

Central adiposity and insulin resistance, both hallmark features of PCOS, emerged as major determinants of periodontal inflammation in our study. Strong positive correlations were found between BMI and waist circumference and periodontal parameters. Furthermore, when we evaluated the clinical parameters involved in these associations, insulin levels were found to be independently associated with probing depth and gingival inflammation and showed a clear discriminative capacity for periodontal disease status, thus reinforcing the close relationship between metabolic dysfunction and periodontal pathology [29]. These findings are consistent with previous evidence identifying central adiposity and IR as pivotal biological links between PCOS and periodontal inflammation [15]. In this line, previous research by our group identified IR as a contributor to periodontitis extension independently of obesity itself, thereby reinforcing the idea that IR plays a central role in the development and extent of periodontal diseases [24].

These observations are consistent with the concept that IR contributes to impaired host defences and exaggerated inflammatory responses within periodontal tissues [3, 9, 11, 24] Mechanistically, defective insulin signalling has been associated with neutrophil dysfunction, increased oxidative stress, and endothelial impairment, all of which can compromise periodontal tissue integrity [9]. In addition, hyperinsulinemia and hyperglycaemia favour the formation of advanced glycation end products and the activation of RAGE-dependent inflammatory pathways, further amplifying local periodontal inflammation [11].

Complement component C3 plays a central role in the pathogenesis of periodontal diseases by amplifying the host inflammatory response to dysbiotic subgingival microbiota [30]. Dysregulated C3 activation further stimulates the production of inflammatory cytokines, including TNF-α and IL1β, and enhances matrix metalloproteinase activity and RANKL-mediated osteoclastogenesis, thereby contributing to connective tissue degradation and alveolar bone loss [30–32]. Moreover, previous evidence has demonstrated a close association between C3c levels and IR [32]. Consistently, the elevated C3c and insulin levels observed in our PCOS population, together with their associations with periodontal parameters, support complement overactivation as a contributor to increased inflammatory burden and a higher prevalence of periodontal diseases in women with PCOS. Gingivitis in PCOS can activate innate immune responses in a proinflammatory and pro-oxidant context, potentially exacerbating systemic inflammation and metabolic dysregulation [27]. In fact, we have recently reported that gingivitis in PCOS can activate innate immune responses within a proinflammatory and pro-oxidant context, potentially exacerbating systemic inflammation and metabolic dysregulation [23]. In the present study, we observed an increase in C3 and insulin in the PCOS group, which confirms the overactivation of innate immunity observed in this syndrome and an increased risk of PDs (OR = 5.36).

On the other hand, we saw that high HDL levels were correlated with lower periodontal inflammation. These results are consistent with recent studies demonstrating a reverse relationship between inflammatory cytokines and HDL [33, 34]. The hypothesis is based on the fact that HDL contains antioxidant enzymes and proteins, whereas periodontal pathogens impair HDL’s antioxidant function [33, 34].

The integration of periodontal inflammation into the pathophysiological framework of PCOS highlights the systemic nature of the disorder and underscores the relevance of extra-ovarian inflammatory sites in modulating metabolic dysfunction.

Notably, the associations observed were mainly related to inflammatory periodontal parameters, such as BOP and plaque accumulation, rather than to irreversible tissue damage. This suggests that metabolic and inflammatory alterations in PCOS primarily influence the early and potentially reversible stages of periodontal disease. Given that recent research has shown an exacerbated alteration of bone metabolism in patients with periodontitis, treating gingivitis in order to reverse gingival inflammation is especially relevant in patients with PCOS. However, the cross-sectional design of this study does not allow causal relationships to be established. Therefore, the observed associations should be interpreted with caution. Longitudinal and interventional studies with long-term follow-up are needed to further investigate the temporal and causal relationship between PCOS and periodontal disease.

### Clinical implications

The present findings highlight the importance of periodontal assessment in women with PCOS and suggest that metabolic control, particularly targeting insulin resistance, can have beneficial effects on periodontal health.

## Conclusion

Overall, our findings suggest that PCOS is independently associated with periodontal diseases and that this association is mediated by metabolic and inflammatory alterations, with insulin resistance emerging as a key link between systemic and oral health.

## Data Availability

The data that support the findings of this study are available on request from the corresponding author. The data are not publicly available due to privacy or ethical restrictions.

## Abbreviations

ANOVA: Analysis of Variance
BMI: Body Mass Index
BOP: Bleeding on Probing
C3c: Complement Factor C3
CAL: Clinical Attachment Loss
CHAID: Chi-square Automatic Interaction Detection
DHEAS: Dehydroepiandrosterone Sulfate
FSH: Follicle-Stimulating Hormone
GCF: Gingival crevicular fluid
HDLc: High-Density Lipoprotein Cholesterol
HOMA-IR: Homeostasis Model Assessment of Insulin Resistance
hsCRP: High-Sensitivity C-Reactive Protein
LH: Luteinizing Hormone
LDLc: Low-Density Lipoprotein Cholesterol
OR: Odds Ratios
PCOS: Polycystic Ovary Syndrome
PDs: Periodontal Diseases
PPD: Probing Pocket Depth
RBP4: Retinol-Binding Protein 4
SHBG: Sex Hormone-Binding Globulin
TC: Total Cholesterol
TG: Triglycerides
17OH: 17-Hydroxyprogesterone (17-OH Progesterone)
WHO: World Health Organization
WHR: Waist-to-Hip Ratio
WSR: Waist-to-Stature Ratio

## References

[1] Sanz M, Marco del Castillo A, Jepsen S, Gonzalez-Juanatey JR, D’Aiuto F, Bouchard P, et al. Periodontitis and cardiovascular diseases: Consensus report. J Clin Periodontol. 2020;47(3):268–88. 10.1111/JCPE.13189. PMC7027895 32011025

[2] Dietrich T, Sharma P, Walter C, Weston P, Beck J. The epidemiological evidence behind the association between periodontitis and incident atherosclerotic cardiovascular disease. J Clin Periodontol. 2013;40(14):S70–84. 10.1111/JCPE.12062. Suppl 14(SUPPL. 23627335

[3] Song IS, Han K, Park YM, Ji S, Jun SH, Ryu JJ, et al. Severe periodontitis is associated with insulin resistance in non-abdominal obese adults. J Clin Endocrinol Metab. 2016;101(11):4251–9. 10.1210/jc.2016-2061. 27598510

[4] Benguigui C, Bongard V, Ruidavets JB, Chamontin B, Sixou M, Ferrières J, et al. Metabolic syndrome, insulin resistance, and periodontitis: a cross-sectional study in a middle-aged French population. J Clin Periodontol. 2010;37(7):601–8. 10.1111/j.1600-051X.2010.01571. 20492076

[5] Márquez-Arrico CF, Silvestre-Rangil J, Gutiérrez-Castillo L, Martinez-Herrera M, Silvestre FJ, Rocha M. Association between Periodontal Diseases and Polycystic Ovary Syndrome: A Systematic Review. J Clin Med. 2020;9(5). 10.3390/JCM9051586. PMC7290429 32456146

[6] Machado V, Escalda C, Proença L, Mendes JJ, Botelho J. Is There a Bidirectional Association between Polycystic Ovarian Syndrome and Periodontitis? A Systematic Review and Meta-analysis. J Clin Med. 2020;9(6):1–16. 10.3390/JCM9061961. PMC7355910 32585861

[7] WHO. Síndrome del ovario poliquístico. 2025. Available from: https://www.who.int/es/news-room/fact-sheets/detail/polycystic-ovary-syndrome. Cited 22 Sep 2025.

[8] Bañuls C, Rovira-Llopis S, Martinez de Marañon A, Veses S, Jover A, Gomez M, et al. Metabolic syndrome enhances endoplasmic reticulum, oxidative stress and leukocyte–endothelium interactions in PCOS. Metabolism. 2017;71:153–62. 10.1016/j.metabol.2017.02.012. 28521868

[9] Victor VM, Rocha M, Bañuls C, Alvarez A, De Pablo C, Sanchez-Serrano M, et al. Induction of oxidative stress and human leukocyte/endothelial cell interactions in polycystic ovary syndrome patients with insulin resistance. J Clin Endocrinol Metab. 2011;96(10):3115–22. 10.1210/JC.2011-0651. 21778215

[10] Jepsen S, Caton JG, Albandar JM, Bissada NF, Bouchard P, Cortellini P, et al. Periodontal manifestations of systemic diseases and developmental and acquired conditions: Consensus report of workgroup 3 of the 2017 World Workshop on the Classification of Periodontal and Peri-Implant Diseases and Conditions. J Periodontol. 2018;89:S237–48. 10.1002/JPER.17-0733. 29926943

[11] Zhang Q, Yang Z, Ou X, Zhang M, Qin X, Wu G. The role of immunity in insulin resistance in patients with polycystic ovary syndrome. Front Endocrinol (Lausanne). 2025;15. 10.3389/FENDO.2024.1464561. PMC11797073 39911236

[12] Porwal S, Tewari S, Sharma RK, Singhal SR, Narula SC. Periodontal Status and High-Sensitivity C-Reactive Protein Levels in Polycystic Ovary Syndrome With and Without Medical Treatment. J Periodontol. 2014;85(10):1380–9. 10.1902/jop.2014.130756. 24592911

[13] Özçaka Ö, Buduneli N, Ceyhan BO, Akcali A, Hannah V, Nile C, et al. Is Interleukin-17 Involved in the Interaction Between Polycystic Ovary Syndrome and Gingival Inflammation? J Periodontol. 2013;84(12):1827–37. 10.1902/jop.2013.120483. 23327719

[14] Pavankumar S, Yellarthi PK, Sandeep JN, Boyapati R, Damera TK, Naveen VKG. Evaluation of periodontal status in women with polycystic ovary syndrome versus healthy women: a cross-sectional study. J Yeungnam Med Sci. 2023;40(Suppl):S17–22. 10.12701/JYMS.2023.00143. PMC10718611 37157778

[15] Liu X, Wang F, Wang X, Luan Q. Impact of body mass index and polycystic ovary syndrome (PCOS) subtypes on periodontal health in Chinese women with PCOS and periodontitis. Gynecol Endocrinol. 2024;40(1):2405097. 10.1080/09513590.2024.2405097. 39305479

[16] Deepti TDS, Chander Narula DS, Rani Singhal DS, Kumar Sharma DR. Effect of Non-surgical Periodontal Therapy Along With Myo-Inositol on High Sensitivity C-Reactive Protein and Insulin Resistance in Polycystic Ovary Syndrome Women Having Chronic Periodontitis: A Randomized Controlled Trial. J Periodontol. 2017;88(10):999–1011. 10.1902/jop.2017.170121. 28598285

[17] Rotterdam ESHRE, ASRM-Sponsored PCOS consensus workshop group. Revised 2003 consensus on diagnostic criteria and long-term health risks related to polycystic ovary syndrome (PCOS). Hum Reprod. 2004;19(1):41–7. 10.1093/humrep/deh098. 14688154

[18] World Health Organization. Oral health surveys: basic methods – 5th edition. Geneva; 2013. Available from: https://www.who.int/publications/i/item/9789241548649. Cited 4 May 2026.

[19] Papapanou PN, Sanz M, Buduneli N, Dietrich T, Feres M, Fine DH et al. Periodontitis: Consensus report of workgroup 2 of the 2017 World Workshop on the Classification of Periodontal and Peri-Implant Diseases and Conditions. Vol. 89. 2018;89:S173–82. 10.1002/JPER.17-0721 29926951

[20] Silness J, Löe H. Periodontal disease in pregnancy. ii. correlation between oral hygiene and periodontal condtion. Acta Odontol Scand. 1964;22(1):121–35. 10.3109/00016356408993968. 14158464

[21] Ramfjord SP. The Periodontal Disease Index (PDI). J Periodontol. 1967;38(6):602–10. 10.1902/JOP.1967.38.6.602. 5237683

[22] Pelechá-Salvador M, Márquez-Arrico CF, Fernández-Reyes M, Perea-Galera L, Hermenejildo J, Morillas C, et al. Autophagy and mitophagy in circulating immune cells of women with polycystic ovary syndrome: A cardiovascular perspective. Free Radic Biol Med. 2025;241:161–70. 10.1016/J.FREERADBIOMED.2025.09.025. 40957527

[23] Márquez-Arrico CF, Pelechá-Salvador M, Fernández-Reyes M, Silvestre FJ, Perea-Galera L, Hermenejildo J, et al. Gingival Inflammation Modulates NLRP3 Inflammasome Signalling in Peripheral Blood Mononuclear Cells of PCOS Patients: A Case-Control Study. Antioxid (Basel). 2025;14(5). 10.3390/ANTIOX14050507. PMC12108338 40427389

[24] Martinez-Herrera M, Silvestre FJ, Silvestre-Rangil J, Bañuls C, Rocha M, Hernández-Mijares A. Involvement of insulin resistance in normoglycaemic obese patients with periodontitis: A cross-sectional study. J Clin Periodontol. 2017;44(10):981–8. 10.1111/JCPE.12773. 28696512

[25] Dou Y, Xin J, Zhou P, Tang J, Xie H, Fan W, et al. Bidirectional association between polycystic ovary syndrome and periodontal diseases. Front Endocrinol (Lausanne). 2023;14. 10.3389/fendo.2023.1008675. PMC9899846 36755917

[26] Bitonti V, Perri T, Cigni L, Familiari D, Vazzana G, Franco R. The Association Between Periodontal Disease and Polycystic Ovary Syndrome: A Systematic Review and Meta-Analysis. Dent J (Basel). 2025;13(5). 10.3390/dj13050188. PMC12110398 40422608

[27] Márquez-Arrico CF, Silvestre FJ, Fernández-Reyes M, López-Domènech S, Hermenejildo J, Abad-Jiménez Z, et al. Gingival inflammation and leukocyte-endothelium cell interactions in women with polycystic ovary syndrome. J Periodontol. 2025;96(5):478–89. 10.1002/JPER.24-0148. PMC12123394 39403884

[28] Genco RJ, Borgnakke WS. Risk factors for periodontal disease. Periodontol 2000. 2013;62(1):59–94. 10.1111/J.1600-0757.2012.00457.X. 23574464

[29] Wzatek M, Bahammam S, Buiga P, Haddad K, Sima C. Oral Neutrophil Free Fatty Acid Receptors Expression May Link Oral Host and Microbiome Lipid Metabolism. Front oral health. 2022;3:821326. 10.3389/FROH.2022.821326. PMC8937037 35320973

[30] Hajishengallis G, Hasturk H, Lambris JD, Apatzidou DA, Belibasakis GN, Bostanci N, et al. C3-targeted therapy in periodontal disease: moving closer to the clinic. Trends Immunol. 2021;42(10):856–64. 10.1016/j.it.2021.08.001. PMC8487962 34483038

[31] Li J, Xu Z, Ayre WN, Liu X. AMY-101 as complement C3 inhibitor for periodontitis therapy: mechanisms, efficacy, and clinical translation. Front Immunol. 2025;16. 10.3389/FIMMU.2025.1587126. PMC12069041 40364839

[32] Al-Domi HA, Al Haj Ahmad RM. Association between complement component C3 and body composition: a possible obesity inflammatory biomarker for insulin resistance. Asia Pac J Clin Nutr. 2017;26(6):1082–7. 10.6133/APJCN.012017.02. 28917234

[33] Nepomuceno R, Pigossi SC, Finoti LS, Orrico SRP, Cirelli JA, Barros SP, et al. Serum lipid levels in patients with periodontal disease: A meta-analysis and meta-regression. J Clin Periodontol. 2017;44(12):1192–207. 10.1111/jcpe.12792. 28782128

[34] Ehteshami A, Shirban F, Bagherniya M, Sathyapalan T, Jamialahmadi T, Sahebkar A. The Association between High-density Lipoproteins and Periodontitis. Curr Med Chem. 2024;31(39):6407–28. 10.2174/0929867331666230726140736. 37493158

